# Inferring Horizontal Gene Transfer

**DOI:** 10.1371/journal.pcbi.1004095

**Published:** 2015-05-28

**Authors:** Matt Ravenhall, Nives Škunca, Florent Lassalle, Christophe Dessimoz

**Affiliations:** 1 University College London, London, United Kingdom; 2 ETH Zurich, Zurich, Switzerland; 3 Swiss Institute of Bioinformatics, Zurich, Switzerland; 4 European Molecular Biology Laboratory, European Bioinformatics Institute, Wellcome Trust Genome Campus, Hinxton, Cambridge, United Kingdom; University of Toronto, Canada

## Abstract

Horizontal or Lateral Gene Transfer (HGT or LGT) is the transmission of portions of genomic DNA between organisms through a process decoupled from vertical inheritance. In the presence of HGT events, different fragments of the genome are the result of different evolutionary histories. This can therefore complicate the investigations of evolutionary relatedness of lineages and species. Also, as HGT can bring into genomes radically different genotypes from distant lineages, or even new genes bearing new functions, it is a major source of phenotypic innovation and a mechanism of niche adaptation. For example, of particular relevance to human health is the lateral transfer of antibiotic resistance and pathogenicity determinants, leading to the emergence of pathogenic lineages [1]. Computational identification of HGT events relies upon the investigation of sequence composition or evolutionary history of genes. Sequence composition-based ("parametric") methods search for deviations from the genomic average, whereas evolutionary history-based ("phylogenetic") approaches identify genes whose evolutionary history significantly differs from that of the host species. The evaluation and benchmarking of HGT inference methods typically rely upon simulated genomes, for which the true history is known. On real data, different methods tend to infer different HGT events, and as a result it can be difficult to ascertain all but simple and clear-cut HGT events.

*This is a 'Topic Page' article for PLOS Computational Biology*.

## Introduction

Horizontal gene transfer (HGT) was first observed in 1928, in Frederick Griffith’s experiment. Showing that virulence was able to pass from virulent to nonvirulent strains of *Streptococcus pneumoniae*, Griffith demonstrated that genetic information can be horizontally transferred between bacteria via a mechanism known as transformation [2]. Similar observations in the 1940s [3] and 1950s [4] showed evidence that conjugation and transduction are additional mechanisms of horizontal gene transfer [5].

**Fig 1:**
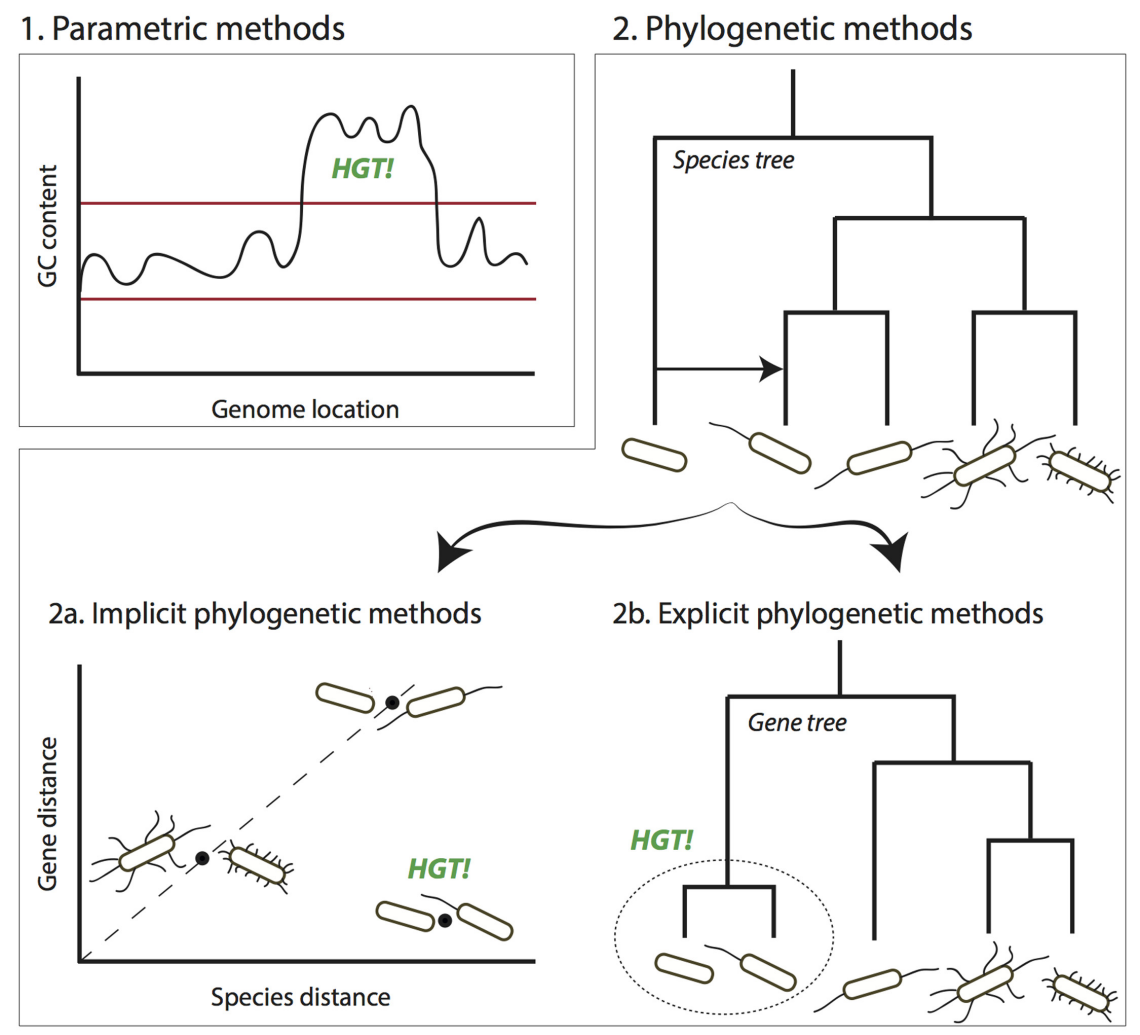
Conceptual overview of HGT inference methods. (1) Parametric methods infer HGT by computing a statistic, here GC content, for a sliding window and comparing it to the typical range over the entire genome, here indicated between the two red horizontal lines. Regions with atypical values are inferred as having been horizontally transferred. (2) Phylogenetic approaches rely on the differences between genes and species tree evolution that result from HGT. Explicit phylogenetic methods reconstruct gene trees and infer the HGT events likely to have resulted into that particular gene tree. Implicit phylogenetic methods bypass gene tree reconstruction, e.g., by looking at discrepancies between pairwise distances between genes and their corresponding species.

To infer HGT events, which may not necessarily result in phenotypic changes, most contemporary methods are based on analyses of genomic sequence data. These methods can be broadly separated into two groups: parametric and phylogenetic methods (Fig 1). Parametric methods search for sections of a genome that significantly differ from the genomic average, such as guanine-cytosine (GC) content or codon usage [6]. Phylogenetic methods examine evolutionary histories of genes involved and identify conflicting phylogenies. Phylogenetic methods can be further divided into those that reconstruct and compare phylogenetic trees explicitly and those that use surrogate measures in place of the phylogenetic trees [7].

The main feature of parametric methods is that they only rely on the genome under study to infer HGT events that may have occurred on its lineage. It has been a considerable advantage at the early times of the sequencing era, when few closely related genomes were available for comparative methods. However, because they rely on the uniformity of the host's signature to infer HGT events, not accounting for the host's intragenomic variability will result in overpredictions—flagging native segments as possible HGT events [8]. Similarly, the transferred segments need to exhibit the donor's signature and to be significantly different from the recipient's [6]. Furthermore, genomic segments of foreign origin are subject to the same mutational processes as the rest of the host genome, and so the difference between the two tends to vanish over time, a process referred to as amelioration [9]. This limits the ability of parametric methods to detect ancient HGTs.

Phylogenetic methods benefit from the recent availability of many sequenced genomes. Indeed, as for all comparative methods, phylogenetic methods can integrate information from multiple genomes and in particular integrate them using a model of evolution. This lends them the ability to better characterize the HGT events they infer—notably by designating the donor species and time of the transfer. However, models have limits and need to be used cautiously. For instance, the conflicting phylogenies can be the result of events not accounted for by the model, such as unrecognized paralogy due to duplication followed by gene losses. Also, many approaches rely on a reference species tree that is supposed to be known, when in many instances it can be difficult to obtain a reliable species tree. Finally, the computational costs of reconstructing many gene and species trees can be prohibitively expensive. Phylogenetic methods tend to be applied to genes or protein sequences as basic evolutionary units, which limits their ability to detect HGT in regions outside or across gene boundaries.

Because of their complementary approaches—and often nonoverlapping sets of HGT candidates—combining predictions from parametric and phylogenetic methods can yield a more comprehensive set of HGT candidate genes. Indeed, combining different parametric methods has been reported to significantly improve the quality of predictions [10,11]. Moreover, in the absence of a comprehensive set of true horizontally transferred genes, discrepancies between different methods [12,13] might be resolved through combining parametric and phylogenetic methods. However, combining inferences from multiple methods also entails a risk of an increased false positive rate [14].

## Parametric Methods

**Fig 2:**
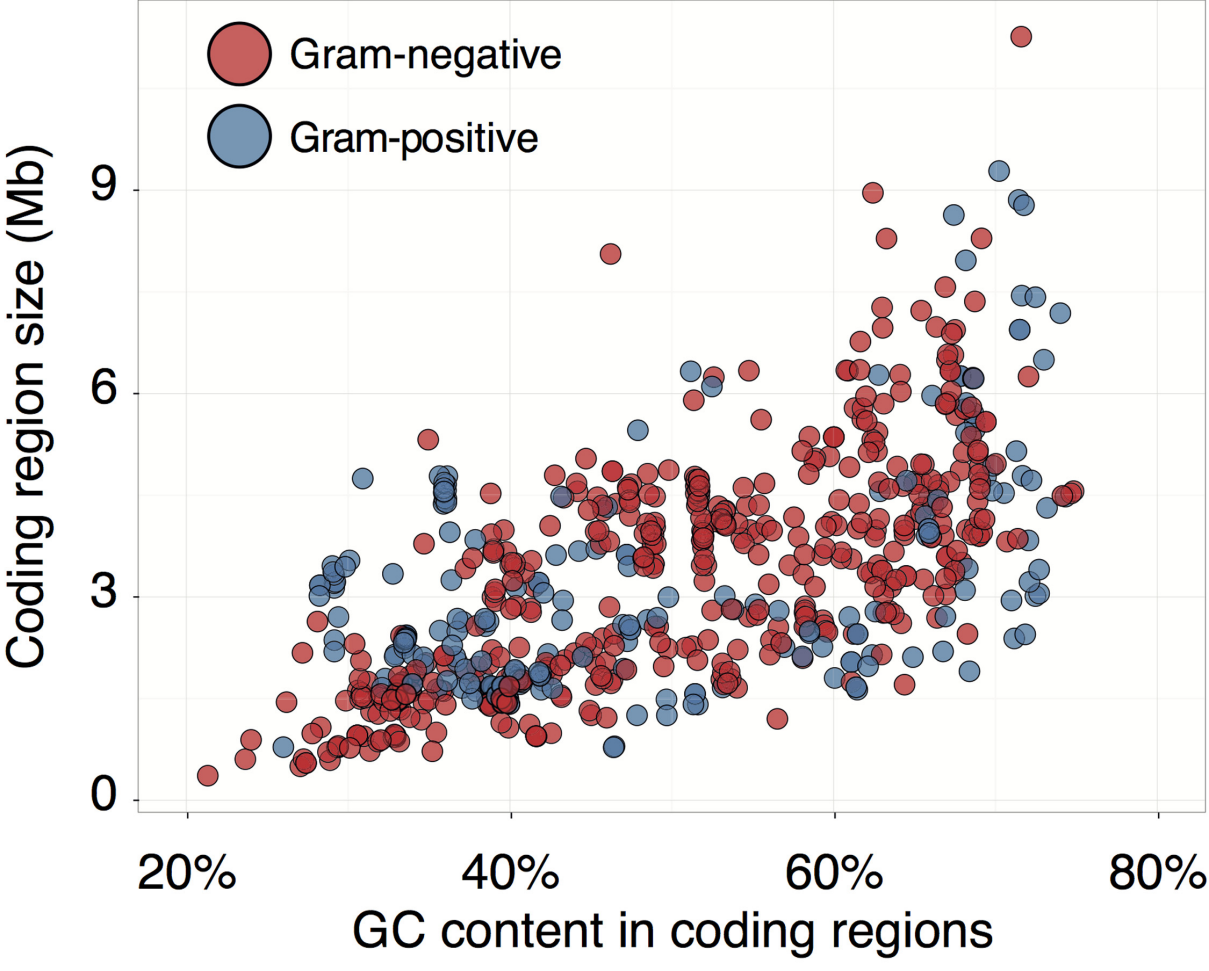
Average GC content of coding regions compared to the genome size for selected bacteria. There is considerable variation in average GC content across species, which makes it relevant as a genomic signature.

Parametric methods to infer HGT use characteristics of the genome sequence specific to particular species or clades, also called genomic signatures. If a fragment of the genome strongly deviates from the genomic signature, this is a sign of a potential horizontal transfer. For example, because bacterial GC content falls within a wide range (see Fig 2), GC content of a genome segment is a simple genomic signature. Commonly used genomic signatures include nucleotide composition [15], oligonucleotide frequencies [16], or structural features of the genome [17].

To detect HGT using parametric methods, the host's genomic signature needs to be clearly recognizable. However, the host's genome is not always uniform with respect to the genome signature; for example, GC content of the third codon position is lower close to the replication terminus [18], and GC content tends to be higher in highly-expressed genes [19]. Not accounting for such intragenomic variability in the host can result in overpredictions, flagging native segments as HGT candidates [8]. Larger sliding windows can account for this variability at the cost of a reduced ability to detect smaller HGT regions [12].

Just as importantly, horizontally transferred segments need to exhibit the donor's genomic signature. This might not be the case for ancient transfers where transferred sequences are subjected to the same mutational processes as the rest of the host genome, potentially causing their distinct signatures to "ameliorate" [9] and become undetectable through parametric methods. For example, *Bdellovibrio bacteriovorus*, a predatory δ-Proteobacterium, has homogeneous GC content, and it might be concluded that its genome is resistant to HGT [20]. However, subsequent analysis using phylogenetic methods identified a number of ancient HGT events in the genome of *B*. *bacteriovorus* [21]. Similarly, if the inserted segment was previously ameliorated to the host's genome, as is the case for prophage insertions [22], parametric methods might miss predicting these HGT events. Also, the donor's composition must significantly differ from the recipient's to be identified as abnormal, a condition that might be missed in the case of short- to medium-distance HGT, which are the most prevalent. Furthermore, it has been reported that recently acquired genes tend to be more AT-rich than the recipient's average [15], which indicates that differences in GC content signature may result from unknown postacquisition mutational processes rather than from the donor's genome.

### Nucleotide composition

Bacterial GC content falls within a wide range, with *Candidatus Zinderia insecticola* having a GC content of 13.5% [23] and *Anaeromyxobacter dehalogenans* having a GC content of 75% [24] (see Fig 2). Even within a closely related group of α-Proteobacteria, values range from approximately 30% to 65% [25]. These differences can be exploited when detecting HGT events as a significantly different GC content for a genome segment can be an indication of foreign origin [15] (see Fig 2).

### Oligonucleotide spectrum

The oligonucleotide spectrum (or k-mer frequencies) measures the frequency of all possible nucleotide sequences of a particular length in the genome. It tends to vary less within genomes than between genomes and therefore can also be used as a genomic signature [26]. A deviation from this signature suggests that a genomic segment might have arrived through horizontal transfer.

The oligonucleotide spectrum owes much of its discriminatory power to the number of possible oligonucleotides; if n is the size of the vocabulary and w is oligonucleotide size, the number of possible distinct oligonucleotides is n^w^; for example, there are 4^5^ = 1,024 possible pentanucleotides. Some methods can capture the signal recorded in motifs of variable size [27], thus capturing both rare and discriminative motifs along with frequent but more common ones.

Codon usage bias, a measure related to codon frequencies, was one of the first detection methods used in methodical assessments of HGT [16]. This approach requires a host genome which contains a bias towards certain synonymous codons (different codons which code for the same amino acid), which is clearly distinct from the bias found within the donor genome. The simplest oligonucleotide used as a genomic signature is the dinucleotide; for example, the third nucleotide in a codon and the first nucleotide in the following codon represent the dinucleotide least restricted by amino acid preference and codon usage [28].

It is important to optimise the size of the sliding window in which to count the oligonucleotide frequency; a larger sliding window will better buffer variability in the host genome at the cost of being worse at detecting smaller HGT regions [29]. A good compromise has been reported using tetranucleotide frequencies in a sliding window of 5 kb with a step of 0.5 kb [30].

A convenient method of modelling oligonucleotide genomic signatures is to use Markov chains. The transition probability matrix can be derived for endogenous versus acquired genes [31], from which Bayesian posterior probabilities for particular stretches of DNA can be obtained [32].

### Structural features

Just as the nucleotide composition of a DNA molecule can be represented by a sequence of letters, its structural features can be encoded in a numerical sequence. The structural features include interaction energies between neighbouring base pairs [33], the angle of twist that makes two bases of a pair noncoplanar [34], or DNA deformability induced by the proteins shaping the chromatin [35].

The autocorrelation analysis of some of these numerical sequences show characteristic periodicities in complete genomes [36]. In fact, after detecting archaea-like regions in the thermophilic bacteria *Thermotoga maritima* [37], periodicity spectra of these regions were compared to the periodicity spectra of the homologous regions in the archaea *Pyrococcus horikoshii* [17]. The revealed similarities in the periodicity were strong supporting evidence for a case of massive HGT between the bacteria and the archaea kingdoms [17].

### Genomic context

The existence of genomic islands, short (typically 10–200 kb long) regions of a genome which have been acquired horizontally, lends support to the ability to identify non-native genes by their location in a genome [38]. For example, a gene of ambiguous origin which forms part of a non-native operon could be considered to be non-native. Alternatively, flanking repeat sequences or the presence of nearby integrases or transposases can indicate a non-native region [39]. A machine-learning approach combining oligonucleotide frequency scans with context information was reported to be effective at identifying genomic islands [40]. In another study, the context was used as a secondary indicator, after removal of genes which are strongly thought to be native or non-native through the use of other parametric methods [10].

## Phylogenetic Methods

The use of phylogenetic analysis in the detection of HGT was advanced by the availability of many newly sequenced genomes. Phylogenetic methods detect inconsistencies in gene and species evolutionary history in two ways: explicitly, by reconstructing the gene tree and reconciling it with the reference species tree, or implicitly, by examining aspects that correlate with the evolutionary history of the genes in question, e.g., patterns of presence and absence across species, or unexpectedly short or distant pairwise evolutionary distances.

### Explicit phylogenetic methods

The aim of explicit phylogenetic methods is to compare gene trees with their associated species trees. While weakly-supported differences between gene and species trees can be due to inference uncertainty, statistically significant differences can be suggestive of HGT events (see Fig 1A). For example, if two genes from different species share the most recent ancestral connecting node in the gene tree, but the respective species are spaced apart in the species tree, an HGT event can be invoked. Such an approach can produce more detailed results than parametric approaches because the involved species, time, and direction of transfer can potentially be identified.

As discussed in more details below, phylogenetic methods range from simple methods merely identifying discordance between gene and species trees to mechanistic models inferring probable sequences of HGT events. An intermediate strategy entails deconstructing the gene tree into smaller parts until each matches the species tree (genome spectral approaches).

Explicit phylogenetic methods rely upon the accuracy of the input rooted gene and species trees, yet these can be challenging to build [41]. Even when there is no doubt in the input trees, the conflicting phylogenies can be the result of evolutionary processes other than HGT, such as duplications and losses, causing these methods to erroneously infer HGT events when paralogy is the correct explanation. Similarly, in the presence of incomplete lineage sorting, explicit phylogeny methods can erroneously infer HGT events [42]. That is why some explicit model-based methods test multiple evolutionary scenarios involving different kinds of events and compare their fit to the data, given parsimonious or probabilistic criteria.

#### Tests of topologies

To detect sets of genes that fit poorly to the reference tree, one can use statistical tests of topology, such as the Kishino-Hasegawa (KH) [43], Shimodaira-Hasegawa (SH) [44], and Approximately Unbiased (AU) [45] tests. These tests assess the likelihood of the gene sequence alignment when the reference topology is given as the null hypothesis.

The rejection of the reference topology is an indication that the evolutionary history for that gene family is inconsistent with the reference tree. When these inconsistencies cannot be explained using a small number of nonhorizontal events, such as gene loss and duplication, an HGT event is inferred.

One such analysis checked for HGT in groups of homologs of the γ-Proteobacterial lineage [46]. Six reference trees were reconstructed using either the highly conserved small subunit ribosomal RNA sequences, a consensus of the available gene trees or concatenated alignments of orthologs. The failure to reject the six evaluated topologies, and the rejection of seven alternative topologies, was interpreted as evidence for a small number of HGT events in the selected groups.

Tests of topology identify differences in tree topology taking into account the uncertainty in tree inference, but they make no attempt at inferring how the differences came about. To infer the specifics of particular events, genome spectral or subtree pruning and regraft methods are required.

#### Genome spectral approaches

In order to identify the location of HGT events, genome spectral approaches decompose a gene tree into substructures (such as bipartitions or quartets) and identify those that are consistent or inconsistent with the species tree.

Removing one edge from a reference tree produces two unconnected subtrees, each containing a disjoint set of nodes—a bipartition. If a bipartition is present in both the gene and the species trees, it is compatible; otherwise, it is conflicting. These conflicts can indicate an HGT event or may be the result of uncertainty in gene tree inference. To reduce uncertainty, bipartition analyses typically focus on strongly supported bipartitions such as those associated with branches with bootstrap values or posterior probabilities above certain thresholds. Any gene family found to have one or several conflicting, but strongly supported, bipartitions is considered as an HGT candidate [47,48].

Alternatively, trees can be decomposed into quartets. Quartets are trees consisting of four leaves. In bifurcating (fully resolved) trees, each internal branch induces a quartet whose leaves are either subtrees of the original tree or actual leaves of the original tree. If the topology of a quartet extracted from the reference species tree is embedded in the gene tree, the quartet is compatible with the gene tree. Conversely, incompatible strongly supported quartets indicate potential HGT events [49]. Quartet mapping methods are much more computationally efficient and naturally handle heterogeneous representation of taxa among gene families, making them a good basis for developing large-scale scans for HGT, looking for highways of gene sharing in databases of hundreds of complete genomes [50,51].

#### Subtree pruning and regrafting

A mechanistic way of modelling an HGT event on the reference tree is to first cut an internal branch—i.e., prune the tree—and then regraft it onto another edge, an operation referred to as subtree pruning and regrafting (SPR) [52]. If the gene tree was topologically consistent with the original reference tree, the editing results in an inconsistency. Similarly, when the original gene tree is inconsistent with the reference tree, it is possible to obtain a consistent topology by a series of one or more prune and regraft operations applied to the reference tree. By interpreting the edit path of pruning and regrafting, HGT candidate nodes can be flagged and the host and donor genomes inferred [48,53]. To avoid reporting false positive HGT events due to uncertain gene tree topologies, the optimal "path" of SPR operations can be chosen among multiple possible combinations by considering the branch support in the gene tree. Weakly supported gene tree edges can be ignored a priori [54], or the support can be used to compute an optimality criterion [55,56].

Because conversion of one tree to another by a minimum number of SPR operations is NP-Hard [57], solving the problem becomes considerably more difficult as more nodes are considered. The computational challenge lies in finding the optimal edit path, i.e., the one that requires the fewest steps [58,59], and different strategies are used in solving the problem. For example, the HorizStory algorithm reduces the problem by first eliminating the consistent nodes [60]; recursive pruning and regrafting reconciles the reference tree with the gene tree and optimal edits are interpreted as HGT events. The SPR methods included in the supertree reconstruction package SPRSupertrees substantially decrease the time of the search for the optimal set of SPR operations by considering multiple localised subproblems in large trees through a clustering approach [61].

#### Model-based reconciliation methods

Reconciliation of gene and species trees entails mapping evolutionary events onto gene trees in a way that makes them concordant with the species tree, given a mechanistic model. Different reconciliation models exist, differing in the types of event they consider to explain the incongruences between gene and species tree topologies. Early methods exclusively modelled horizontal transfers (T) [52,55]. More recent ones also account for duplication (D), loss (L), incomplete lineage sorting (ILS), or homologous recombination (HR) events. The difficulty is that by allowing for multiple types of events, the number of possible reconciliations increases rapidly. For instance, conflicting gene tree topologies might be explained in terms of a single HGT event or multiple duplication and loss events. Both alternatives can be considered plausible reconciliation depending on the frequency of these respective events along the species tree.

Reconciliation methods can rely on a parsimonious or a probabilistic framework to infer the most likely scenario(s), where the relative cost and probability of D, T, and L events can be fixed a priori or estimated from the data [62]. The space of DTL reconciliations and their parsimony costs—which can be extremely vast for large multicopy gene family trees—can be efficiently explored through dynamic programming algorithms [63–65]. In some programs, the gene tree topology can be refined where it was uncertain to fit a better evolutionary scenario as well as the initial sequence alignment [63,66,67]. More refined models account for the biased frequency of HGT between closely related lineages [68], reflecting the loss of efficiency of HR with phylogenetic distance [69], for ILS [70], or for the fact that the actual donor of most HGT belong to extinct or unsampled lineages [71]. Further extensions of DTL models are being developed towards an integrated description of the genome evolution processes. In particular, some of them consider horizontal transfer at multiple scales—modelling independent evolution of gene fragments [72] or recognising coevolution of several genes (e.g., due to cotransfer) within and across genomes [73].

### Implicit phylogenetic methods

In contrast to explicit phylogenetic methods, which compare the agreement between gene and species trees, implicit phylogenetic methods compare evolutionary distances or sequence similarity. Here, an unexpectedly short or long distance from a given reference compared to the average can be suggestive of an HGT event (see Fig 1). Because tree construction is not required, implicit approaches tend to be simpler and faster than explicit methods.

However, implicit methods can be limited by disparities between the underlying correct phylogeny and the evolutionary distances considered. For instance, the most similar sequence as obtained by the highest-scoring BLAST hit is not always the evolutionarily closest one [74].

#### Top sequence match in a distant species

A simple way of identifying HGT events is by looking for high-scoring sequence matches in distantly related species. For example, an analysis of the top BLAST hits of protein sequences in the bacteria *Thermotoga maritima* revealed that most hits were in archaea rather than closely-related bacteria, suggesting extensive HGT between the two [37]; these predictions were later supported by an analysis of the structural features of the DNA molecule [17].

However, this method is limited to detecting relatively recent HGT events. Indeed, if the HGT occurred in the common ancestor of two or more species included in the database, the closest hit will reside within that clade, and therefore the HGT will not be detected by the method. Thus, the threshold of the minimum number of foreign top BLAST hits to observe to decide a gene was transferred is highly dependent on the taxonomic coverage of sequence databases. Therefore, experimental settings may need to be defined in an ad-hoc way [75].

#### Discrepancy between gene and species distances

The molecular clock hypothesis posits that homologous genes evolve at an approximately constant rate across different species [76]. If one only considers homologous genes related through speciation events (referred to as “orthologous" genes), their underlying tree should by definition correspond to the species tree. Therefore, assuming a molecular clock, the evolutionary distance between orthologous genes should be approximately proportional to the evolutionary distances between their respective species. If a putative group of orthologs contains xenologs (pairs of genes related through an HGT), the proportionality of evolutionary distances may only hold among the orthologs, not the xenologs [77].

Simple approaches compare the distribution of similarity scores of particular sequences and their orthologous counterparts in other species; HGT are inferred from outliers [78,79]. The more sophisticated DLIGHT (Distance Likelihood-based Inference of Genes Horizontally Transferred) method considers simultaneously the effect of HGT on all sequences within groups of putative orthologs [7]: if a likelihood-ratio test of the HGT hypothesis versus a hypothesis of no HGT is significant, a putative HGT event is inferred. In addition, the method allows inference of potential donor and recipient species and provides an estimation of the time since the HGT event.

#### Phylogenetic profiles

A group of orthologous or homologous genes can be analysed in terms of the presence or absence of group members in the reference genomes; such patterns are called phylogenetic profiles [80]. To find HGT events, phylogenetic profiles are scanned for an unusual distribution of genes. Isolated occurrence of a gene, i.e., absence of a homolog in other members of a group of closely related species is an indication that the examined gene might have arrived via an HGT event. For example, the three facultatively symbiotic *Frankia* spp. strains are of strikingly different sizes: 5.43 Mbp, 7.50 Mbp, and 9.04 Mbp, depending on their range of hosts [81]. Marked portions of strain-specific genes were found to have no significant hit in the reference database and were possibly acquired by HGT transfers from other bacteria. Similarly, three phenotypically diverse *Escherichia coli* strains (uropathogenic, enterohemorrhagic, and benign) shared about 40% of the total combined gene pool, with the other 60% being strain-specific genes and, consequently, HGT candidates [82]. Further evidence for these genes resulting from HGT was their strikingly different codon usage patterns from the core genes and a lack of gene order conservation (order conservation is typical of vertically-evolved genes) [82]. The presence and absence of homologs (or their effective count) can thus be used by programs to reconstruct the most likely evolutionary scenario along the species tree. Just as with reconciliation methods, this can be achieved through parsimonious [83] or probabilistic estimation of the number of gain and loss events [84,85]. Models can be complexified by adding processes, like the truncation of genes [86], but also by modelling the heterogeneity of rates of gain and loss across lineages [87] and/or gene families [85,88].

#### Clusters of polymorphic sites

Genes are commonly regarded as the basic units transferred through an HGT event. However, it is also possible for HGT to occur within genes. For example, it has been shown that horizontal transfer between closely related species results in more exchange of ORF fragments [89,90], a type a transfer called gene conversion, mediated by homologous recombination. The analysis of a group of four *E*. *coli* and two *Shigella flexneri* strains revealed that the sequence stretches common to all six strains contain polymorphic sites, consequences of homologous recombination [91]. Clusters of excess of polymorphic sites can thus be used to detect tracks of DNA recombined with a distant relative [92]. This method of detection is, however, restricted to the sites in common with all analysed sequences, limiting the analysis to a group of closely related organisms.

## Evaluation

The existence of the numerous and varied methods to infer HGT raises the question of how to validate individual inferences and of how to compare the different methods.

A main problem is that, as with other types of phylogenetic inferences, the actual evolutionary history cannot be established with certainty. As a result, it is difficult to obtain a representative test set of HGT events. Furthermore, HGT inference methods vary considerably in the information they consider and often identify inconsistent groups of HGT candidates [6,93]; it is not clear to what extent taking the intersection, the union, or some other combination of the individual methods affects the false positive and false negative rates [14].

Parametric and phylogenetic methods draw on different sources of information; it is therefore difficult to make general statements about their relative performance. Conceptual arguments can, however, be invoked. While parametric methods are limited to the analysis of single genomes or pairs of genomes, phylogenetic methods provide a natural framework to take advantage of the information contained in multiple genomes. In many cases, segments of genomes inferred as HGT based on their anomalous composition can also be recognised as such on the basis of phylogenetic analyses or through their mere absence in genomes of related organisms. In addition, phylogenetic methods rely on explicit models of sequence evolution, which provide a well-understood framework for parameter inference, hypothesis testing, and model selection. This is reflected in the literature, which tends to favour phylogenetic methods as the standard of proof for HGT [94–97]. The use of phylogenetic methods thus appears to be the preferred standard, especially given that the increase in computational power coupled with algorithmic improvements has made them more tractable [61,71], and that the ever denser sampling of genomes lends more power to these tests.

Considering phylogenetic methods, several approaches to validating individual HGT inferences and benchmarking methods have been adopted, typically relying on various forms of simulation. Because the truth is known in simulation, the number of false positives and the number of false negatives are straightforward to compute. However, simulating data does not trivially resolve the problem, because the true extent of HGT in nature remains largely unknown, and specifying rates of HGT in the simulated model is always hazardous. Nonetheless, studies involving the comparison of several phylogenetic methods in a simulation framework could provide quantitative assessment of their respective performances and thus help the biologist in choosing objectively proper tools [56].

Standard tools to simulate sequence evolution along trees such as INDELible [98] or PhyloSim [99] can be adapted to simulate HGT. HGT events cause the relevant gene trees to conflict with the species tree. Such HGT events can be simulated through subtree pruning and regrafting rearrangements of the species tree [54]. However, it is important to simulate data that are realistic enough to be representative of the challenge provided by real datasets, and simulation under complex models are thus preferable. A model was developed to simulate gene trees with heterogeneous substitution processes in addition to the occurrence of transfer and accounting for the fact that transfer can come from now extinct donor lineages [100]. Alternatively, the genome evolution simulator Artificial Life Simulator (ALF) [101] directly generates gene families subject to HGT by accounting for a whole range of evolutionary forces at the base level but in the context of a complete genome. Given simulated sequences which have HGT, analysis of those sequences using the methods of interest and comparison of their results with the known truth permits study of their performance. Similarly, testing the methods on sequences known not to have HGT enables the study of false positive rates.

Simulation of HGT events can also be performed by manipulating the biological sequences themselves. Artificial chimeric genomes can be obtained by inserting known foreign genes into random positions of a host genome [12,102–104]. The donor sequences are inserted into the host unchanged or can be further evolved by simulation [7], e.g., using the tools described above.

One important caveat to simulation as a way to assess different methods is that simulation is based on strong simplifying assumptions that may favour particular methods [105].

## Supporting Information

S1 Text Version history of the text file. (XML)

S2 Text Peer reviews and response to reviews. Human-readable versions of the reviews and authors' responses are available as comments on this article. (XML)
